# The Effect of Pre-Exercise Hyperhydration on Exercise Performance, Physiological Outcomes and Gastrointestinal Symptoms: A Systematic Review

**DOI:** 10.1007/s40279-023-01885-2

**Published:** 2023-07-25

**Authors:** William T. Jardine, Brad Aisbett, Monica K. Kelly, Louise M. Burke, Megan L. Ross, Dominique Condo, Julien D. Périard, Amelia J. Carr

**Affiliations:** 1 School of Exercise and Nutrition Sciences, Centre for Sport Research, Deakin University, 221 Burwood Highway, Burwood, VIC 3125 Australia; 2 Mary MacKillop Institute for Health Research, Exercise and Nutrition Research Program, Australian Catholic University, Watson, ACT 2602 Australia; 3 Research Institute for Sport and Exercise, University of Canberra, Bruce, ACT 2617 Australia

## Abstract

**Background:**

Fluid loss during prolonged exercise in hot conditions poses thermoregulatory and cardiovascular challenges for athletes that can lead to impaired performance. Pre-exercise hyperhydration using nutritional aids is a strategy that may prevent or delay the adverse effects of dehydration and attenuate the impact of heat stress on exercise performance.

**Objectives:**

The aim of this systematic review was to examine the current literature to determine the effect of pre-exercise hyperhydration on performance, key physiological responses and gastrointestinal symptoms.

**Methods:**

English language, full-text articles that compared the intervention with a baseline or placebo condition were included. An electronic search of Medline Complete, SPORTDiscus and Embase were used to identify articles with the final search conducted on 11 October 2022. Studies were assessed using the American Dietetic Association Quality Criteria Checklist.

**Results:**

Thirty-eight studies involving 403 participants (*n* = 361 males) were included in this review (*n* = 22 assessed exercise performance or capacity). Two studies reported an improvement in time-trial performance (range 5.7–11.4%), three studies reported an improvement in total work completed (kJ) (range 4–5%) and five studies reported an increase in exercise capacity (range 14.3–26.2%). During constant work rate exercise, nine studies observed a reduced mean heart rate (range 3–11 beats min^−1^), and eight studies reported a reduced mean core temperature (range 0.1–0.8 °C). Ten studies reported an increase in plasma volume (range 3.5–12.6%) compared with a control. Gastrointestinal symptoms were reported in 26 studies, with differences in severity potentially associated with factors within the ingestion protocol of each study (e.g. treatment, dose, ingestion rate).

**Conclusions:**

Pre-exercise hyperhydration may improve exercise capacity during constant work rate exercise due to a reduced heart rate and core temperature, stemming from an acute increase in plasma volume. The combination of different osmotic aids (e.g. glycerol and sodium) may enhance fluid retention and this area should continue to be explored. Future research should utilise valid and reliable methods of assessing gastrointestinal symptoms. Furthermore, studies should investigate the effect of hyperhydration on different exercise modalities whilst implementing a strong level of blinding. Finally, females are vastly underrepresented, and this remains a key area of interest in this area.

**Supplementary Information:**

The online version contains supplementary material available at 10.1007/s40279-023-01885-2.

## Key Points

**Table:** 

Pre-exercise hyperhydration appears to acutely increase plasma volume which may reduce heart rate and core temperature during constant work rate exercise to exhaustion compared with control.
Implementing pre-exercise hyperhydration may induce gastrointestinal symptoms during exercise in some individuals, with the severity of symptoms potentially associated with the dose, timing and ingestion rate of the hyperhydration strategy.
The effect of hyperhydration on time-trial performance is equivocal and provides an area for future research.

## Introduction

Hypohydration, the state of being in fluid deficit of ≥ 2% of body mass (BM) [1], has been shown to impair exercise performance in both temperate and hot conditions relative to euhydration (normal body water content) [1–3]. A reduction in total body water and total blood volume contributes to decreased venous return, which influences left ventricle end diastolic volume [4]. Subsequently, a reduction in cardiac output is seen, as stroke volume decreases and heart rate increases [5, 6]. Furthermore, peripheral vasoconstriction occurs to increase total peripheral resistance to maintain mean arterial pressure [4]. During constant work rate exercise in hot conditions (> 26.5 °C, ranging between 36% and 80% $\dot{V}$O_2max_), for every 1% of BM lost there is a concurrent increase in heart rate of ~4 beats min^−1^ [5]. Vasoconstriction, as a result of hypohydration, can also cause a reduction in blood flow to the skin [7]. A decline in skin blood flow can negatively impact the body’s ability to dissipate heat via convection and evaporation, resulting in an increase in heat storage [6, 8, 9]. As such, for each 1% of BM lost, core temperature is suggested to increase by ~0.15 to 0.25 °C during exercise at different intensities in both field and laboratory-based studies [6, 9–12]. In addition to performance impairments [13], a rise in core temperature can lead to heat-related illness, such as heat exhaustion and exertional heat stroke [3, 14].

Pre-exercise hyperhydration (an increase in total body water above that of normal levels) provides a strategy to delay or reduce the adverse effects of exercise-induced hypohydration [15]. Hyperhydration aims to provide a small, but potentially useful, fluid excess to offset some of the sweat loss that cannot be compensated for by fluid intake during exercise in hot conditions [16–18]. Ingesting large amounts of fluid alone (e.g. water) is not an effective method to induce hyperhydration as it inhibits the release of anti-diuretic hormone (ADH; also referred to as vasopressin or arginine vasopressin [19]) and increases urine production [20, 21]. Therefore, nutritional aids with an osmotic capacity, such as glycerol and sodium, have been investigated for their ability to increase total body water status prior to exercise [18, 22].

Glycerol, a three-carbon alcohol, is an osmotic agent that has been used previously by athletes in hyperhydration protocols [23]. Glycerol can be found naturally in food (e.g. soybeans) and as an additive to manufactured foods due to its properties as a sweetener and thickening agent. Once ingested, it is absorbed in the gastrointestinal tract and is then evenly distributed in the intra- and extracellular compartments.[24]. As glycerol is a solute, it creates an osmotic gradient which promotes the flow of fluids to the area of increased solute concentration to maintain equilibrium [24]. Glycerol may also promote fluid balance via a renal effect that leads to reduced urine production [24, 25], independent of the hormonal responses (i.e. ADH and aldosterone) typically associated with fluid retention [19]. In 2010, glycerol was added to the World Anti-Doping Agency’s (WADA) prohibited list due to its classification as a plasma volume expander, which might be used to mask blood doping practices by manipulating the measures used to detect them (e.g. haematocrit and haemoglobin) [26]. However, in 2018, after further investigation showed that any effect on these parameters was minor [27], glycerol was removed from this list and its potential for use by athletes in hyperhydration protocols was reinstated.

During the period in which athletes were prohibited from deliberately consuming glycerol, the continued interest in hyperhydration focused on the role of sodium as an osmolyte [22, 28–30]. Sodium is a positively charged ion (Na^+^) that is the main contributor to plasma osmolality [31] and plays a crucial role in determining the flow of fluids in the intra- and extracellular compartments [24]. An acute increase in sodium intake via various compounds (e.g. chloride and citrate) or modalities of ingestion (e.g. dissolved in solution and tablet form) in combination with fluid has been utilised to induce hyperhydration [15, 29, 30]. When ingested, sodium stimulates ADH secretion, promoting water reabsorption in the kidney and reducing urine output [32]. Indeed, the greater osmotic and renal effects of sodium appear to make it more effective in retaining a co-ingested fluid bolus than observed with glycerol hyperhydration [30], potentially due to its direct effect on the renal system, compared with glycerol [19], and the influence of sodium on plasma osmolality.

Pre-exercise glycerol-induced hyperhydration strategies have been shown to attenuate the fluid deficit during exercise in the heat, with benefits including a reduction in heart rate and core temperature during constant work rate exercise [17, 33, 34]. However, both glycerol- and sodium-induced hyperhydration may induce several adverse side effects, including gastrointestinal symptoms such as nausea and diarrhoea [17, 35, 36]. These symptoms may counterbalance any potential performance benefits associated with an improved hydration status. Gastrointestinal issues are common in endurance athletes, with incidence dependent on the duration and intensity of exercise [37]. Furthermore, compared with temperate conditions, exercising in hot environments has been shown to increase the severity and incidence of gastrointestinal symptoms [38]. Gastrointestinal symptoms can cause a reduction in athletic performance and, in some serious cases, withdrawal from competition [39]. Further investigation of the effect of hyperhydration on the severity of exercise-induced gastrointestinal symptoms is warranted.

Many sporting competitions are to be hosted in hot environments (e.g. the 2024 Olympic Games, Paris) where hyperhydration may act as a valuable preparation strategy for competition in prolonged events. However, the current evidence that pre-exercise hyperhydration enhances sports performance is equivocal. Indeed, the last systematic review published on this topic was published in 2007, prior to glycerol being placed on the banned list [40]. Therefore, the aim of this review is to systematically analyse the existing evidence on pre-exercise hyperhydration with regards to exercise performance and capacity, gastrointestinal symptoms and key physiological measures prior to and during exercise.

## Methodology

### Search Strategy

This systematic review was completed in line with the preferred reporting items for systematic reviews and meta-analysis (PRISMA) statement [41] and registered via PROSPERO (2022CRD42022299305). An electronic search of Medline Complete and SPORTDiscus (via the EBSCOHost database) and Embase was used to identify peer-reviewed, human studies available in the English language with no date restrictions up until the final search which was conducted on 11 October 2022. An online systematic review management system (Covidence Systematic Review Software, Melbourne, Australia) was used to house all studies for subsequent screening. The full search strategy for each database can be found in Tables S2, S3 and S4 of the Electronic Supplementary Material (ESM). In this review, the term ‘record’ refers to an entire publication and the term ‘study’ refers to each individual study within a single record.

### Eligibility Criteria

Studies were included if the experimental condition (i.e. hyperhydrating treatment) was compared with baseline or a placebo condition. Where the protocol involved exercise, the participants had to be classified as ‘Performance Level Two (PL2): Recreational Athletes’ (training at least three times per week and for at least 5 h per week) as a minimum for both males and females, according to an established participant classification framework [42, 43] for the record to be included. Studies that involved hyperhydration during a protocol without exercise required participants to be defined as healthy, active and free from any medical condition. Studies that did not contain an exercise component were included as they provided information regarding gastrointestinal symptoms and some physiological outcomes. All legal methods (as outlined by WADA) of inducing hyperhydration were included (i.e. intravenous methods were excluded as they have no relevance to the sporting population) [44]. Studies were excluded if they involved concurrent interventions (e.g. combining hyperhydration with another heat mitigation strategy), if hyperhydration was not initially induced or if the hydration protocol comprised an initial dehydration period and then rehydration protocol prior to exercise. Conference abstracts, review papers, unpublished theses and papers that were not published in the English language were also excluded. Titles and abstracts were initially screened for relevance to hyperhydration, and potential articles were further screened after accessing the full text. Screening was completed by two authors (WTJ, MKK) and conflicts were discussed and resolved between four authors (WTJ, MKK, AJC, DC).

### Outcome Variables

All included studies were coded by one author (WTJ) for the following: sample size (*n*), gender (M, F), age (y), body mass (kg), $\dot{V}$O_2max_ or V̇O_2peak_ (mL kg^−1^ min^−1^), training status, exercise mode, exercise distance (km), exercise intensity (% lactate threshold (LT), %$\dot{V}$O_2max_, %$\dot{V}$O_2peak_, %*W*_max_, %WR_max_), exercise duration (min), environmental temperature (°C), relative humidity (RH: %), air flow (m s^−1^), hyperhydrating treatment and dose, fluid dose, hyperhydration strategy, changes in plasma volume (%), sweat rate (L h^−1^), core and skin temperature (°C), urine volume (mL), fluid retention (where possible – calculated as fluid ingested minus urine volume; mL), gastrointestinal symptoms, exercise performance (i.e. time-trial; TT) and exercise capacity (i.e. time to exhaustion; TTE). Where data was only presented using figures, extraction was completed using online software (https://automeris.io/WebPlotDigitizer/). Mean differences and 95% confidence intervals were calculated using the reported mean, standard deviation (SD) and sample size.

### Quality Assessment

Studies included in this review were assessed for quality using the American Dietetic Association Quality Criteria Checklist [45]. This tool assesses each individual study for bias in recruitment of participants, study design, methodology, statistical analyses and reporting of results. Two authors assessed each individual study (WTJ and MKK), and any discrepancies were resolved after discussion between the two authors. The quality assessment score for each study can be found in Table S1 in the ESM.

## Results

### Literature Search

**Fig. 1:**
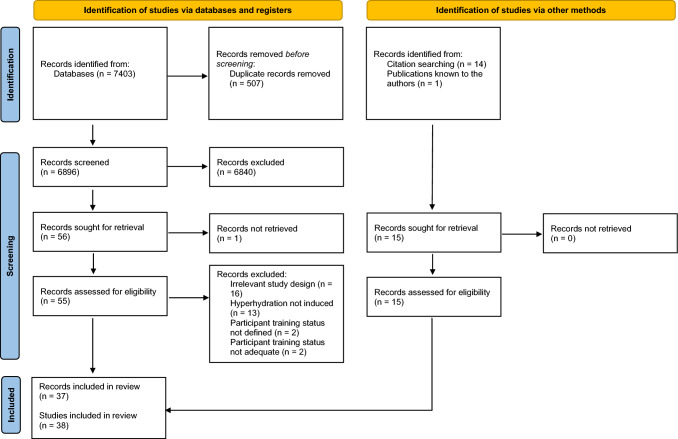
PRISMA flowchart demonstrating the identification, screening and inclusion of studies for this review [41]

The original search yielded 7403 records, of which 507 were identified as duplicates. Fifteen records were identified through hand searching of reference lists from key, original investigations or records known to the authors [15, 29, 30, 46]. Title and abstract screening excluded 6825 records, principally because the study failed to involve the methodology (inducement of hyperhydration) or participant pool of interest (i.e. below Performance Level 2 if the included an exercise protocol). Seventy-one records were assessed for eligibility from examination of the full text, 34 records were excluded due to non-relevant study design or methodology (e.g. including a rehydration period), participant training status or the lack of access to the full text. The final review included 37 records, which reported 38 studies (Fig. 1). The study characteristics and methodological approach is presented in Table 1 for studies that involved an exercise performance or exercise capacity component (i.e. time-trial or time to exhaustion) and Table 2 for those that did not (i.e. constant work rate exercise or passive rest). A meta-analytic approach was deemed not feasible as there is very limited consistency between experimental studies, as Tables 1 and 2 outline (i.e. wide variety in the type of hyperhydration agent used, dose of hyperhydration agent and co-ingested fluid bolus, timing between ingestion and exercise, environmental conditions in which exercise was performed in, classification level of participants and dietary standardisation techniques).

**Table 1:** The characteristics of studies that employed an exercise performance or exercise capacity component included in this review

Author and year	Participant characteristics (*n*, sex, age, body mass, population)	Study design	Hyperhydration osmolyte	Fluid amount co-ingested	Elapsed time between completion of hyperhydration and beginning of exercise	Comparator	Exercise protocol and environmental conditions	Dietary standardisation
Anderson et al. 2001 [17]	*n* = 6, M, 23.3 ± 6.6 years, 72.0 ± 4.3 kg, endurance trained	Randomised, double-blind, crossover	1.0 g kg^−1^ BM glycerol	20 mL kg^−1^ BM	120 min	Sweetened beverage	90 min of cycling at 98% LT and 15 min performance test in 35 °C and 30% RH. Air flow not reported	Standardised diet for 24 h prior to trial
Coutts et al. 2002 [18]	*n* = 10, 7 M, 3 F, 33.3 ± 2.3 years, 70.9 ± 3.3 kg, well trained	Randomised, double-blind, crossover	1.2 g kg^−1^ BM glycerol	25 mL kg^−1^ BM	70 min	Carbohydrate electrolyte solution	Olympic distance triathlon in ~30.5 °C WBGT or ~25.4 °C WBGT	Participants instructed on dietary intake for 3 days prior to trial. Standardised meal consumed morning of
Souza et al. 2018 [54]	*n* = 10, M, 40.5 ± 9.7 years, 72.5 ± 8.4, trained runners	Crossover	Fluid containing 2.4 g L^−1^ of NaCl	Ad libitum	Ad libitum consumption 60 min prior to exercise	Water consumed ad libitum	10 km TT in the field in 28 °C	N/A
Dini et al. 2007 [59]	*n* = 14, M, 26 ± 5 years, 88 ± 7 kg, highly trained rowers	Randomised, parallel groups	1.0 g kg^−1^ BM glycerol 1.0 g kg^−1^ BM of glycerol + 1.0 g kg^−1^ BM of glycerol consumed during exercise	28.5 mL kg^−1^ BM 28.5 mL kg^−1^ BM + 4.5 mL kg^−1^ BM	180 min	Water	Incremental rowing test in 36 °C and 30% RH	Food and fluid diary completed and analysed
Easton et al. 2007 [55]	*n* = 23, M, ~31 years ~75.0 kg, endurance trained	Randomised, parallel groups	7-day supplementation 2.0 g kg^−1^ BM glycerol and/or 11.4 g creatine	1000 mL		Pre-supplementation	40 min of cycling at 63% W_max_ and a 16.1 km TT in ~30 °C, ~70% RH and air flow 1.8 m s^−1^	Food and fluid dairy completed and analysed
Gigou et al. 2012 [28]	*n* = 6, M, 31 ± 7 years, 78 ± 10 kg, highly trained	Randomised, crossover	7.5 g L^−1^ NaCl	26 mL kg^−1^ BM	50 min	No Treatment	18 km treadmill running TT in ~28 °C and ~30% RH	Food and fluid log kept for 24 h prior to trial
Goulet et al. 2002 [63]	*n* = 1, M, 27 years, 72.2 kg, highly trained	Randomised, double-blind, crossover, case study	1.2 g kg^−1^ BM glycerol	26 mL kg^−1^ BM	40 min	Sweetened beverage	120 min of cycling at 66.8% $\dot{V}$O_2max_ followed by an incremental TTE in 25 °C and ~40% RH	Food and fluid log kept for 24 h prior to trial
Goulet et al. 2006 [62]	*n* = 6, M, 25.2 years, 68.5 kg, endurance trained	Randomised, double-blind, crossover	1.2 g kg^−1^ BM glycerol	26 mL kg^−1^ BM	40 min	Sweetened beverage	120 min of cycling at 65% $\dot{V}$O_2max_ followed by an incremental test to exhaustion in 25 °C and ~40% RH	Fluid and diet log kept for 24 and 48 h prior to each trial. Verbal confirmation upon arrival
Goulet et al. 2008 [47]	*n* = 6, 5 M, 1 F, 36.5 ± 5.5 years, 68.3 ± 4.9 kg, endurance trained	Randomised, crossover	1.2 g kg^−1^ BM glycerol	26 mL kg^−1^ BM	10 min	Sweetened Beverage	120 min of cycling at 65% $\dot{V}$O_2max_ followed by an incremental TTE in ~27 °C and 55% RH. Air flow included but not reported	Food and fluid log kept for 24 h prior to trial. Standardised breakfast and fluid before trial
Hillman et al. 2013 [60]	*n* = 7, M, 28 ± 8 years, 73.2 ± 9.6 kg, well trained	Randomised, crossover	1.2 g kg^−1^ BM glycerol	26 mL kg^−1^ BM	90 min	Sweetened beverage No treatment	90 min cycling TT in 35 °C and 40% RH	Food and fluid log kept for 24 h prior to trial
Hitchins et al. 1999 [58]	*n* = 8, M, 27 ± 4.2 years, 73.9 ± 7.4 kg, well-trained	Double-blind, crossover	1.0 g kg^−1^ BM glycerol	22 mL kg^−1^ BM	120 min	Carbohydrate electrolyte solution	30 min of cycling at ~78% $\dot{V}$O_2peak_, 30 min of cycling at a variable PO in ~33 °C, ~58% RH and air flow 5.5 m s^−1^	CHO was provided (8 g kg^−1^ BM). Instructed to consume set volumes of fluid at selected times. Record sheets were completed to confirm this
Kilduff et al. 2004 [61]	*n* = 21, M, ~ 27 years, ~72.0 kg, endurance trained	Randomised, double-blind, parallel groups	7-day of 20 g day^−1^ of creatine + 35 g day^−1^ of glucose	2000 mL		Pre-supplementation	Cycling TTE at ~ 63% $\dot{V}$O_2max_ in ~30 °C, ~70% RH and air flow 3.6 m s^−1^	Normal diet followed. All food and drink weighed and analysed for energy intake and macronutrient content. Caffeine eliminated from diet. Verbal compliance assessed
Marino et al. 2003 [48]	*n* = 7, 6 M, 1 F, 21.2 ± 2.4 years, 78.8 ± 5.1 kg, moderately trained	Randomised, double-blind, crossover	1.2 g kg^−1^ BM glycerol	21 mL kg^−1^ BM	150 min	Placebo	60 min of cycling with 6 × 1 min sprints every 10 min in ~36 °C, ~63% RH and air flow 3.0 m s^−1^	Participants instructed to consume their regular diet and repeat a similar food intake 24 h before each trial
McCullagh et al. 2013 [49]	*n* = 6, 5 M, 1 F, ~69.7 kg, recreationally trained	Cross-over	1.2 g kg^−1^ BM glycerol	26 mL kg^−1^ BM	30 min	Water	10 km run (constant work rate), 40 km cycle (constant work rate), 5 km run (TT) in ~30 °C	Food and fluid log kept for 24 h prior to trial
Montner et al. 1996 Study 1 [34]	*n* = 11, M, 32.5 ± 2.7 years, 67.1 ± 3.5 kg, well trained	Double-blind, crossover	1.2 g kg^−1^ BM glycerol	26 mL kg^−1^ BM	60 min	Placebo	Cycling TTE at ~61% W_max_ in ~24 °C, ~26% RH and air flow 2.03 m s^−1^	Identical self-selected diets were given during both admissions
Montner et al. 1996 Study 2 [34]	*n* = 7, 5 M 2 F, Males 32.6 ± 3.1 years, 72.8 ± 2.2 kg, Females 33.0 ± 7.0 years, 53.5 ± 0.5 kg, well trained	Double-blind, crossover	1.2 g kg^−1^ BM glycerol	26 mL kg^−1^ BM	60 min	Placebo	Cycling TTE at ~60% W_max_ in ~24 °C, ~26% RH and air flow 2.03 m s^−1^	Participants instructed to consume similar diets before each trial
Morris et al. 2015 [22]	*n* = 9, M, 27 ± 4 years, 73.8 ± 9.6 kg, trained	Randomised, double-blind, crossover	60 mg kg^−1^ BM of NaCl	2 mL kg^−1^ BM + unlimited access	120 min	Placebo No treatment	60 min cycling at 50% PO at $\dot{V}$O_2max_ and a 200 kJ TT in 30 °C and ~20% RH	No high sodium foods for 48 h prior to trial. High CHO meal and 1 L water night before
Polyviou et al. 2012 [56]	*n* = 18, M, ~ 31.0 y, 78 ± 8 kg, trained	Randomised, double-blind, parallel groups	7-day supplementation period of 20 g day^−1^ creatine + 2.0 g kg^−1^ BM per day of glycerol + 150 g day^−1^ of glucose or 20 g day^−1^ creatine + 2.0 g kg^−1^ BM per day of glycerol + 100 g day^−1^ of glucose + 1.0 g day^−1^ of alpha-lipoic acid	Not specified	60 min	Pre-supplementation	40 min of cycling at 70% $\dot{V}$O_2max_ followed by a 16.1 km TT in ~30 °C, ~70% RH and air flow 1.8 m s^−1^	Participants completed 7-day food diaries
Scheadler et al. 2010 [53]	*n* = 6, M, 27.8 ± 6.0 years, 69.0 ± 9.2 kg, well trained	Randomised, double-blind, crossover	1.2 g kg^−1^ BM glycerol	26 mL kg^−1^ BM	30 min	Placebo	60 min of treadmill running in ~30 °C and ~50% RH	Food and fluid log kept for 3 days prior to trial
Sims et al. 2007 [16]	*n* = 8, M, 36 ± 11 years, 75.2 ± 6.7 kg, trained	Double-blind, crossover	7.72 g L^−1^ of sodium citrate + 4.5 g L^−1^ of NaCl	10 mL kg^−1^ BM	45 min	0.58 g L^−1^ NaCl	Treadmill running TTE at 70% $\dot{V}$O_2max_ in 32 °C, 50% RH and air flow 1.5 m s^−1^	Same meal (participants choice) was consumed the evening before each testing session. Standardised breakfast consumed before test
Sims et al. 2007 [50]	*n* = 13, F, 26 ± 6 years, 60.8 ± 5 kg, trained	Double-blind, crossover	7.72 g L^−1^ of sodium citrate + 4.5 g L^−1^ of NaCl	10 mL kg^−1^ BM	20 min	0.58 g L^−1^ NaCl	Cycling TTE at 70% $\dot{V}$O_2peak_ in 32 °C, 50% RH and air flow 4.5 m s^−1^	Same meal (participants choice) was consumed the evening before each testing session. Standardised breakfast consumed before test
Wingo et al. 2004 [57]	*n* = 12, M, 24.5 ± 1.1 years, 76.9 ± 1.9 kg, trained	Randomised, double-blind, crossover	1.0 g kg^−1^ BM of glycerol	~ 29.1 mL kg^−1^ BM	35 min	Water No treatment	48 km mountain bike TT (3 × 16 km loops) in ~28 °C	N/A

*n *number, *M* male, *F* female, *kg* kilograms, *y* years, *g kg*^*−1*^* BM* grams per kilogram of body mass, *mL kg*^*−1*^* BM* millilitres per kilogram of body mass, *LT* lactate threshold, *RH* relative humidity, *WBGT* wet-bulb globe temperature, *g L*^*−1*^ grams per litre, *NaCl* sodium chloride, *TT* time-trial, *TTE* time to exhaustion, *Wmax* maximal workload, *m s*^*−1*^ metres per second, *VO*_*2max*_ maximal oxygen consumption, *VO*_*2peak*_ peak oxygen consumption, *PO* power output, *g day*^*−1*^ grams per day

**Table 2:** The characteristics of studies that did not involve an exercise performance or exercise capacity component included in this review

Author and Year	Participant characteristics	Study design	Hyperhydration osmolyte	Fluid amount co-ingested	Elapsed time between completion of hyperhydration and beginning of exercise	Comparator	Exercise protocol and environmental conditions
Beis et al. 2011 [65]	*n* = 14, M, 24 ± 5 years, 69.5 ± 5.0 kg, trained runners	Before and after	7 days of 10 g L^−1^ of creatine, 1.0 g kg^−1^ BM of glycerol and 75 g L^−1^ of glucose	1000 mL	Final dose 240 min pre-exercise	Pre-supplementation	Treadmill running for 2 × 30 min at 60% $\dot{V}$O_2max_ in ~35 °C and ~69% RH, or 10 °C and ~69% RH
Cian et al. 2000 [66]	*n* = 8, M, 27.4 ± 3.7 years, 72.6 ± 5.9 kg, endurance trained	Crossover	1.1 g kg^−1^ BM glycerol consumed with	21.4 mL kg^−1^ BM	90 min	Fluid only (contained 1.2 g L^−1^ NaCl)	15–20 min arm-crank ergometer at 85% $\dot{V}$O_2max_ in 25 °C and 40% RH
Freund et al. 1995 [19]	*n* = 11, M, 24 ± 2 years, 79.4 ± 2.4 kg, healthy and recreational athletes	Counterbalanced, crossover	1.5 g L^−1^ TBW of glycerol	5 mL L^−1^ TBW with initial bolus + 32 mL L^−1^ TBW	N/A	Water No treatment	N/A
Fujii et al. 2021 [68]	*n* = 9 M, 23.4 ± 3.9 years, 65.9 ± 5.2 kg, healthy	Randomised, partially counterbalanced, crossover	0.7% NaCl 0.7% NaCl + 6% dextrin 0.9% NaCl 0.9% NaCl + 6% dextrin 6% dextrin (n = 3)	16–17 mL kg^−1^ BM	N/A	Water	N/A
Goulet et al. 2018 [15]	*n* = 15, M, 22 ± 4 years, 76 ± 7 years, recreationally active	Randomised, counterbalanced, crossover	7.5 g L^−1^ of NaCl 1.4 g kg^−1^ FFM of glycerol 7.5 g L^−1^ of NaCl + 1.4 g kg^−1^ FFM of glycerol	30 mL kg^−1^ FFM	N/A	Each treatment was compared with other hyperhydration treatments	N/A
Koehler et al. 2014 [27]	*n* = 14, 12 M, 2 F, 27 ± 5.4 years, 73.7 ± 8.1 kg, well trained	Crossover	1.0 g kg^−1^ BM glycerol	25 mL kg^−1^ BM	130 min	Sweetened beverage	90 min of cycling at 60% $\dot{V}$O_2max_ in ~ 21.1 °C and ~ 45.2% RH
Latzka et al. 1997 [35]	*n* = 8, M, 23 ± 6 years, 76 ± 15 kg, trained	Crossover	1.2 g kg^−1^ LBM glycerol	29.1 mL kg^−1^ LBM	30 min	Euhydration	120 min of treadmill running at 45% $\dot{V}$O_2max_ in 35 °C, 45% RH and air flow 1.0 m s^−1^
Lyons et al. 1990 [33]	*n* = 6, 4 M, 2 F, 26.2 ± 1.5 years, 71.1 ± 6.0 kg, recreationally trained	Randomised, crossover	1.0 g kg^−1^ BM glycerol + 0.1 g kg^−1^ BM of glycerol every hour after 2 h	21.4 mL kg^−1^ BM	150 min after initial dose	Sweetened beverage Limited fluid	90 min of treadmill exercise at 60% $\dot{V}$O_2max_ in 42 °C, 25% RH and air flow 1.66 m s^−1^
Melin et al. 2002 [67]	*n* = 8, M, 27.4 ± 3.6 years, 72.6 ± 5.9 kg, healthy	Randomised, crossover	1.1 g kg^−1^ BM glycerol	21.4 mL kg^−1^ BM	N/A	Euhydration	N/A
Montner et al. 1999 [51]	*n* = 6, 4 M, 2 F, 27 ± 3 years, 68 ± 6 kg, well trained	Randomised, double-blind, crossover	1.2 g kg^−1^ BM glycerol	26 mL kg^−1^ BM	60 min	Sweetened beverage	110 min of cycling at ~44% $\dot{V}$O_2max_ in 23.5–24.5 °C and 25–27% RH
O'Brien et al. 2005 [64]	*n* = 7, M, 22 ± 3 years, 78.9 ± 7.8 kg, trained	Crossover	1.5 g L^−1^ TBW glycerol ^1^	5 mL L^−1^ + 32 mL L^−1^ TBW	N/A	Water No treatment	N/A
Riedesel et al. 1987 [52]	*n* = 22, 13 M, 9 F, 65–67 kg, healthy	Randomised, crossover	0.5 g kg^−1^ BM or 1.0 g kg^−1^ BM or 1.5 g kg^−1^ BM of glycerol	21.4 mL kg^−1^ BM	N/A	Placebo	N/A
Savoie et al. 2015 [30]	*n* = 17, M, 21 ± 3 years, 79 ± 10 kg, recreationally trained	Randomised, double-blind, counterbalanced, crossover	1.4 g kg^−1^ of glycerol or 7.45 g L^−1^ of NaCl ingested	30 mL kg^−1^ FFM	N/A	Water	N/A
Savoie et al. 2016 [29]	*n* = 16, M, 21 ± 2 years, 77 ± 9 kg, trained	Randomised, counterbalanced, crossover	7.45 g L^−1^ of NaCl ingested in either solution or using dissolvable tablets or 1.4 g kg^−1^ FFM glycerol	30 mL kg^−1^ FFM	N/A	Each treatment was compared with other hyperhydration treatments	N/A
Siegler et al. 2021 [46]	*n* = 19, 13 M, 6 F, 28.3 ± 4.6 years, ~79.2 kg, healthy	Randomised, single-blind, partially counterbalanced, crossover	7.5 g L^−1^ of either sodium bicarbonate or sodium citrate	25 mL kg^−1^ BM	N/A	Sweetened beverage	N/A
Sugihara et al. 2014 [36]	*n* = 8, M, 22.6 ± 1.4 years, 65.6 ± 2.4 kg, healthy	Randomised, crossover	60, 120 or 180 mmol L^−1^ of sodium	16–17 mL kg^−1^ BM	N/A	Water	N/A

*N *number, *M* male, *F* female, *kg* kilograms, *y* years, *g L*^*−1*^ grams per litre, *VO*_*2max*_ maximal oxygen consumption, *RH* relative humidity, *g kg*^*−1*^* BM* grams per kilogram of body mass, *mL kg*^*−1*^* BM* millilitres per kilogram of body mass, *NaCl* sodium chloride, *TBW* total body water, *mL L*^*−1*^ millilitres per litre, *FFM* fat free mass, *m s*^*−1*^ metres per second, *LBM* lean body mass, *mmol L*^*−1*^ millimoles per litre

### Participant Characteristics

A total of 215 participants were included in studies that involved an exercise performance or capacity component (Table 1; *n* = 22). Male participants made up 90% of this cohort. Five studies included at least one female participant with a primarily male cohort [18, 34, 47–49]. One study included female participants only [50]. For studies that did not involve an exercise performance or capacity component (Table 2; *n* = 16), there were a total of 188 participants. Males represented 89% of this cohort. Five studies included at least one female participant [27, 33, 46, 51, 52] with no study including female participants only.

### Exercise Performance

#### Time-Trial Performance

**Fig. 2:**
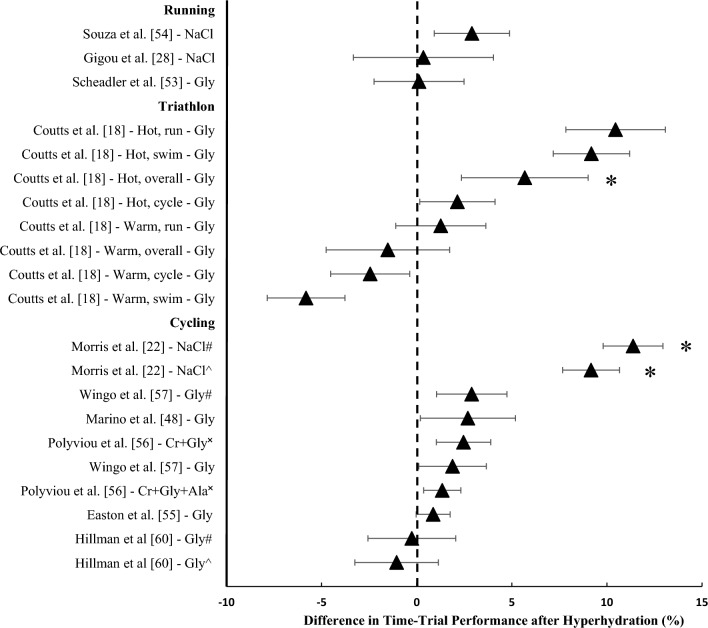
Difference in time-trial performance (%) after hyperhydration compared with control. Data are presented as mean ± 95% CIs. *Significant increase after hyperhydration (*P* < 0.05). *N* = 1 study that did not provide the mean of the performance outcome or variability of the mean [49]. *NaCl* sodium chloride, *Gly* glycerol, *Cr* + *Gly* + *Ala* creatine + glycerol + alpha lipoic acid. ^#^Compared with placebo; ^^^compared with no treatment; ^✖^compared with pre-supplementation

Nine studies investigated time-trial performance after hyperhydration: four studies implemented a running protocol [28, 49, 53, 54], four studies implemented a cycling protocol [22, 55–57] and one study simulated a triathlon [18] (Fig. 2). Of the running studies, three investigated laboratory-based time-trials completed on treadmills, with distances ranging between 5 and 18 km and environmental conditions ranging from 28 °C to 30 °C and 25% to 50% RH, and all found no significant differences between the hyperhydration strategy (glycerol or sodium) and their respective controls [28, 49, 53]. Souza et al. [54] found no significant differences in a field-based 10 km time-trial in 28 °C after sodium ingestion compared with control. One field-based study compared glycerol-induced hyperhydration with a matched fluid-only protocol in two different environmental conditions [hot day: 30.5 °C wet-bulb globe temperature (WBGT); cool day: 25.4 °C WBGT] and found that glycerol hyperhydration improved triathlon time-trial performance by 5.7% compared with placebo on the hotter day [18]. Of the four studies that investigated cycling performance using endurance trained males, three were conducted in laboratory conditions and one in the field. Of the laboratory-based studies, following a 7 day supplementation period of creatine + glycerol [55], or with the addition of glucose + alpha lipoic acid [56], two studies did not find an improvement in exercise performance during a 16.1 km cycling time-trial (~23–27 min) after 40 min of steady-state exercise in ~30 °C and ~70% RH [55, 56]. Comparatively, Morris et al. [22] reported a 9.2% improvement in a 200 kJ performance test (~ 13–15 min) after sodium hyperhydration compared with placebo, and 11.4% improvement compared with no treatment in 30 °C following a 50 min period of steady-state cycling. Wingo et al. [57] reported no significant differences in a 48 km mountain bike time-trial (~158 min) in the field in ~28 °C after glycerol ingestion compared with no treatment or water ingestion.

Three studies reported that hyperhydration improved the rate of energy expenditure (i.e. the total amount of work completed in kJ in an allocated time) over periods ranging from 15 to 89 min [17, 58, 59] (Table 1). Hitchins et al. [58] reported a 5% increase in total work completed after glycerol hyperhydration compared with control during 30 min of variable power output cycling following a 30 min period of steady-state cycling at ~78% $\dot{V}$O_2peak_. A similar result was found by Anderson et al. [17] who reported a ~5% increase in total work completed after glycerol hyperhydration compared with control, during a 15 min cycling performance test in 35 °C and 30% RH following 90 min of cycling at 98% LT. Dini et al. [59] also reported that glycerol-induced hyperhydration improved the work completed at the anaerobic threshold by ~4% in highly trained rowers performing an incremental rowing protocol in 36 °C and 30% RH. Two studies investigated the effect that glycerol-induced hyperhydration had on total distance covered during a set period of time (Table 1). Marino et al. [48] implemented a 60 min cycling protocol in ~35.5 °C, interspersed with short, maximal sprints and found no difference in distance covered compared with placebo (~29.3 km). In agreement with this finding, Hillman et al. [60] also found no difference in total distance covered (~50.5 km) or peak power output (~180 W) during a 90 min cycling protocol in 35 °C and 40% RH compared with water and control.

#### Time to Exhaustion at a Constant Work Rate

**Fig. 3:**
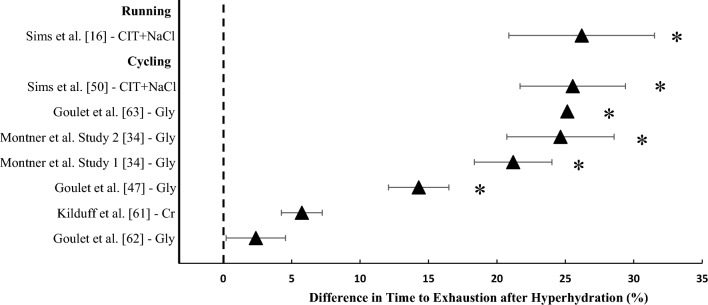
Difference in time to exhaustion (%) after hyperhydration compared with control. Data are presented as mean ± 95% CIs. Study without 95% CIs is a case study. *Significant increase after hyperhydration (*P* < 0.05). *CIT* + *NaCl* sodium citrate + sodium chloride, *Gly* glycerol, *Cr* creatine

Seven studies investigated the effect of hyperhydration on time to exhaustion at a constant work rate (Fig. 3). Two studies found no significant differences between hyperhydration and control [61, 62]. Kilduff et al. [61], implemented a 7 day supplementation period of creatine and found no significant difference in time to exhaustion cycling (~50 min) at 63% $\dot{V}$O_2max_ in 30.3 °C and 70% RH compared with pre-supplementation. Similarly, Goulet et al. [62] reported no difference in time to exhaustion (~12 min) following 120 min of steady-state cycling at 65% $\dot{V}$O_2max_ in 25 °C and ~40% RH between glycerol and placebo. In contrast, five studies found a significant increase in time to exhaustion after glycerol and sodium hyperhydration compared with a control or placebo. An improvement in time to exhaustion (14.3–25.2%) during constant work rate cycling at 60% *W*_max_ and subsequent incremental test was found after glycerol-induced hyperhydration (fluid retention ranging between 53.8% and 79.3%) compared with placebo in temperatures ranging from 23 °C to 27 °C [34, 47, 63]. Two of the aforementioned investigations also reported a concurrent increase in peak power output of 5–8% during the incremental test to exhaustion [47, 63]. Furthermore, two studies reported that a high sodium dose (164 mmol Na^+^ L^−1^; leading to a fluid retention of 44.8%) increased treadmill running time to exhaustion at 70% $\dot{V}$O_2max_ in hot conditions (32 °C, 50% RH) in males by ~26.2% [16] and cycling time to exhaustion at 70% $\dot{V}$O_2peak_ in females by 25.6% compared with a low sodium dose (10 mmol Na^+^ L^−1^; fluid retention data unavailable) [50].

### Gastrointestinal Symptoms

Methods of assessing gastrointestinal symptoms included participants self-reporting [27, 33–36, 48, 49, 53, 55, 56, 59, 61, 62, 64, 65], post-study survey or phone call [17, 18, 58], the Environmental Symptoms Questionnaire [57], a Likert-type scale using ratings of 1–5 for each symptom [15, 28–30, 47, 63] and a modified visual analogue scale [46]. From these methods, 26 of the 38 studies reported that gastrointestinal symptoms were experienced during or following hyperhydration. The symptoms recorded after glycerol ingestion include bloating (severity described as ‘minor’ and subsiding early during exercise) [18, 53, 59], nausea (severity described as ‘minor’, subsiding early during exercise and resulting in participant withdrawal) [18, 35, 53], diarrhoea [17], vomiting [35] and stomach fullness (persisting for 15–20 min) [33]. After a 7 day supplementation period involving glycerol (1.0 g kg^−1^ BM per day), one participant reported gastrointestinal distress [55] and a similar study reported participant withdrawal after gastrointestinal distress [65]. Similarly, a participant withdrew from the study suffering nausea after acute glycerol ingestion [35]. Gastrointestinal distress resulted in participant withdrawal from two studies after a 7 day supplementation period of creatine, glycerol and glucose [56, 65]. However, 13 studies reported no gastrointestinal symptoms after glycerol ingestion [15, 27, 29, 30, 33, 34, 47–49, 58, 62–64]. Six studies did not investigate gastrointestinal symptoms after glycerol ingestion [19, 51, 52, 60, 66, 67].

After sodium ingestion, diarrhoea was reported with prevalence increasing as the dosage of sodium ingested increased [36]. Compared with no treatment, there were no significant differences in ratings of nausea and abdominal bloating after sodium chloride ingestion [28]. There were no significant differences in gastrointestinal symptoms reported in three studies after sodium chloride ingestion when compared with glycerol ingestion [15, 29, 30]. Furthermore, no significant differences were reported when comparing a sodium chloride solution with sodium chloride ingested using dissolvable tablets [29]. A recent study found no difference in gut discomfort when comparing sodium bicarbonate, sodium citrate and a control [46]. Gastrointestinal symptoms were not investigated in five studies involving sodium ingestion [16, 22, 50, 54, 68].

### Physiological Outcomes

#### Heart Rate

**Fig. 4:**
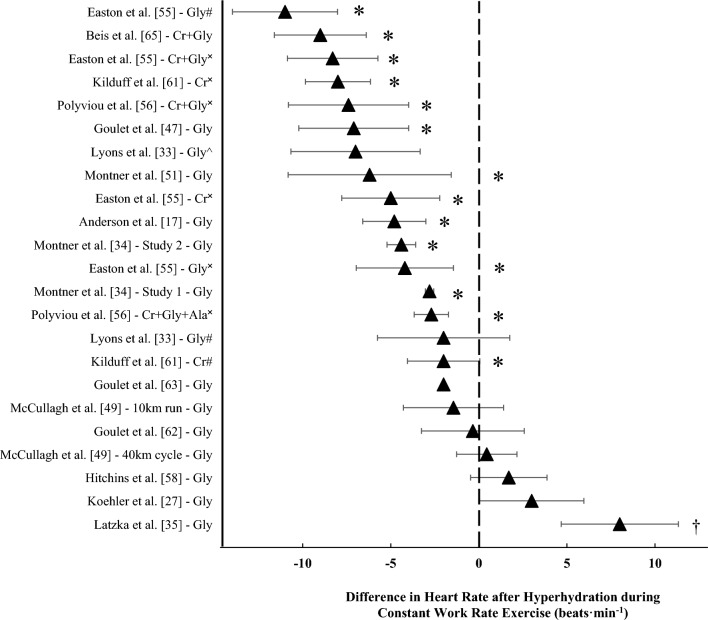
Difference in mean heart rate during constant work rate exercise following hyperhydration compared with control or pre-supplementation. Data are presented as mean ± 95% CIs. Study without 95% CIs is a case study. *Significant decrease reported after hyperhydration (*P* < 0.05). ^†^Significantly higher after hyperhydration (P < 0.05). *Gly* glycerol, *Cr* + *Gly* creatine + glycerol, *Cr* creatine, *Cr* + *Gly* + *Ala* creatine + glycerol + alpha lipoic acid. ^#^Compared with placebo; ^✖^compared with pre-supplementation; ^^^compared with no treatment

During constant work rate exercise (44–70% $\dot{V}$O_2max_, 90% LT and 63% *W*_max_) hyperhydration significantly reduced mean exercising heart rate (3–11 beats min^−1^) in nine studies [17, 34, 47, 50, 55, 56, 61, 65] (Fig. 4). In these studies, cycling protocols were used, except for one which used a running protocol [65]. All studies were conducted in environmental conditions ranging between 23.0 °C and 35.1 °C and 25% and 70% RH. Conversely, seven studies found no significant differences in mean exercising heart rate during constant work rate exercise ranging from 50% to 70% $\dot{V}$O_2max_ and ~78% $\dot{V}$O_2peak_, with one study not reporting the steady-state exercise relative intensity [22, 27, 33, 49, 58, 62, 63] (Fig. 4). Exercise modalities include running, cycling, a combination of running and cycling, and rowing. One study found that after 120 min of treadmill exercise at 45% $\dot{V}$O_2max_ in 35 °C and 45% RH, end-exercise heart rate was ~8 beats min^−1^ higher after glycerol hyperhydration (without additional fluid during exercise) compared with maintaining euhydration during exercise [35]. During variable intensity exercise, hyperhydration significantly increased mean heart rate in one study by 4–5 beats min^−1^, which coincided with an increase in total work by 5% [58]. One study, using a treadmill running time-trial, found a ~5 beats min^−1^ decrease in end-exercise heart rate after hyperhydration compared with control, but this did not translate to a significant performance benefit in a small sample size (*n* = 6) [28]. Five investigations found no significant differences in heart rate during variable intensity exercise [48, 49, 53, 57, 60]

#### Changes in Plasma Volume

**Fig. 5:**
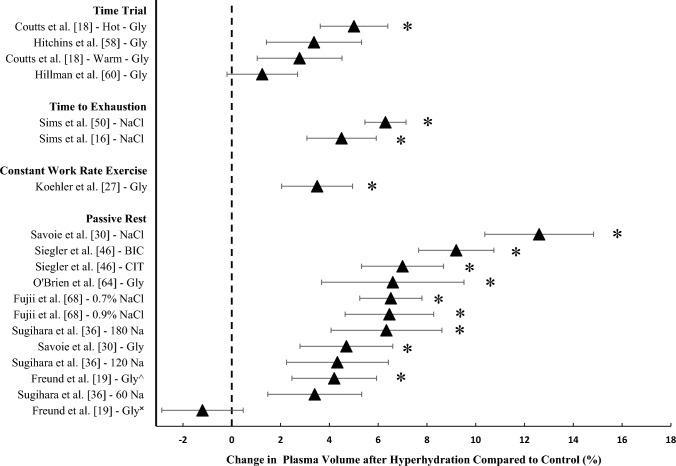
Change in plasma volume (%) following hyperhydration compared with control. Data are presented as mean ± 95% CIs. *Significant increase after hyperhydration (*P* < 0.05). *N* = 4 studies from which % changes at rest and the variability of the mean could not be calculated [33, 35, 56, 65]. *Gly* glycerol, *NaCl* sodium chloride, *BIC* sodium bicarbonate, *CIT* sodium citrate, *Na* sodium. ^^^Compared with no fluid; ^✖^compared with water. Numbers included for Sugihara et al. [36] refer to amount of sodium in millimoles per litre

Hyperhydration significantly increased plasma volume, compared with a control or placebo, in ten studies (13 data points; Fig. 5) [16, 18, 19, 27, 30, 36, 46, 50, 64, 68]. Glycerol-induced hyperhydration, compared with control, significantly increased plasma volume by 3.5–6.6% in five studies [18, 19, 27, 30, 64]. A high sodium dose also significantly increased plasma volume by 4.5–6.3% when compared with a low sodium dosage in two studies prior to constant work rate exercise to exhaustion [16, 50]. Furthermore, consumption of a 0.7% and 0.9% NaCl solution increased plasma volume after 120 min of passive rest by ~6.5% compared with water [68]. A larger dose of sodium (180 mmol L^−1^) significantly increased plasma volume by ~6.3%, compared with water [36]. Sodium chloride ingestion significantly increased plasma volume during passive rest compared with glycerol ingestion by 6.3–7.9% in two studies [29, 30] and by 12.6% compared with water ingestion in one study [30]. Sodium citrate and sodium bicarbonate ingestion increased plasma volume by 7.0% and 9.2%, respectively, after 180 min compared with control (Fig. 5) [46]. Interestingly, only one study measured changes in plasma volume prior to constant work rate exercise and found an increase of 3.5% compared with placebo [27]. All studies calculated changes in plasma volume using the Dill and Costill method of measuring haematocrit and haemoglobin except Goulet et al. [15], who estimated haemoglobin concentration using collected haematocrit values, and O’Brien et al. [64], who estimated plasma volume using radiolabelled albumin. One study measured plasma volume but the results are unavailable due to analytical issues [17].

Hyperhydration, compared with a control, failed to increase plasma volume in nine studies (six data points; Fig. 5). For studies involving glycerol ingestion, no differences were found when comparing glycerol with no fluid or water (haemoglobin and haematocrit values presented only) [33], comparing glycerol with euhydration (% changes at rest not presented) [35], comparing glycerol with placebo [18, 58] or water [19, 60], and glycerol + creatine ingestion compared pre- and post-supplementation (% changes at rest not presented) [56, 65]. Four of these studies are not presented in Fig. 5 due the inability to present the mean changes and provide the variability of the mean [33, 35, 56, 65]. A lower dose of sodium (60 and 120 mmol L^−1^), compared with water, did not enhance plasma volume [36] (Fig. 5).

#### Core and Skin Temperature

**Fig. 6:**
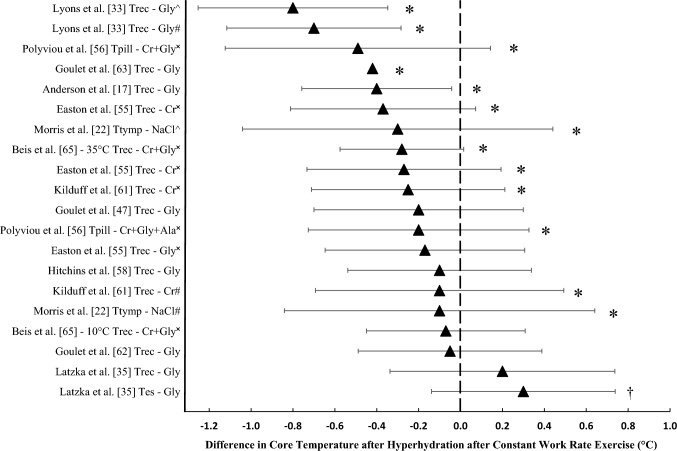
Difference in mean core temperature upon conclusion of constant work rate exercise following hyperhydration, compared with control or pre-supplementation. Data are presented as mean ± 95% CIs. Study without 95% CIs is a case study. *Significant decrease after hyperhydration (*P* < 0.05). ^†^Significant increase after hyperhydration (*P* < 0.05). *Trec* rectal temperature, *Tes* oesophageal temperature, *Tpill* ingestible core temperature monitoring pill, *Ttymp* tympanic temperature, *Gly* glycerol, *Cr* + *Gly* creatine + glycerol, *Cr* creatine, *NaCl* sodium chloride, *Cr* + *Gly* + *Ala* creatine + glycerol + alpha lipoic acid. ^^^Compared with no treatment; ^#^compared with placebo; ^✖^compared with pre-supplementation

There is a lack of agreement on the effect of hyperhydration strategies on core temperature during exercise. In terms of constant work rate exercise, no differences in rectal temperature were found between glycerol and control trials during exercise at 45% $\dot{V}$O_2max_ for 120 min in 35 °C and 45% RH [35]. In agreement with this finding, no differences in core temperature were reported between glycerol and control trials after 30 min of cycling at ~78% $\dot{V}$O_2peak_ in 33.2 °C and 57.8% RH [58] (Fig. 6). Montner et al. [34] found no difference in core temperature after glycerol compared with placebo during a cycling time to exhaustion protocol at 60% *W*_max_ in 23–24.5 °C and 25–27% RH. Furthermore, Goulet et al. [62] reported no difference in core temperature between glycerol and water during 120 min of constant work rate cycling at 65% $\dot{V}$O_2max_ in 25 °C and ~40% RH. The same authors also reported no difference in core temperature between glycerol and placebo during 120 min of constant work rate cycling in 26–27 °C and 55% RH (Fig. 6).

Conversely, an earlier case study of a highly trained triathlete by Goulet et al. [63] reported a 0.42 °C reduction in core temperature after 120 min of constant work rate cycling (66.8% $\dot{V}$O_2max_) in 25 °C and 39% RH. In addition to this, two studies implementing a 90 min exercise protocol at 60% $\dot{V}$O_2max_ and 98% LT, found a 0.4–0.8 °C reduction in core temperature during exercise after glycerol-induced hyperhydration compared with control [17, 33]. Furthermore, a 30 min treadmill run at 60% $\dot{V}$O_2max_ in 35.1 °C and 69.4% RH found a 0.3 °C lower core temperature after a 7 day supplementation period of creatine, glycerol and glucose compared with pre-supplementation, but this finding was not replicated in cool (10 °C) conditions [65] (Fig. 6). Easton et al. [55] also reported a similar finding after a 7 day supplementation protocol of creatine, with a 0.1 °C lower core temperature during 40 min of cycling at 63% WR_max_ in 30 °C and 70% RH compared with pre-supplementation (Fig. 6). Similarly, core temperature was found to be lower (~0.1 °C) after creatine supplementation, at the 35th and 40th min of a constant work rate cycling protocol to exhaustion at 63% $\dot{V}$O_2max_ in 30.3 °C and 70% RH compared with pre-supplementation [61]. After ingestion of creatine + glycerol + glucose only or with the addition of alpha-lipoic acid, one study found that both supplementation groups had a 0.2 °C lower core temperature after 40 min of constant work rate cycling, in 30 °C and 70% RH compared with pre-supplementation values, but no significant differences between supplementation groups [56]. A high sodium dose (164 mmol Na^+^ L^−1^), compared with a low sodium dose (10 mmol Na^+^ L^−1^), significantly reduced core temperature by 0.57 °C in males at exhaustion after treadmill running at 70% $\dot{V}$O_2max_ in 32 °C and 50% RH [16] (Fig. 6). A similar study by the same group found a high sodium beverage significantly lowered the rate of rise of core temperature by 0.4 °C h^−1^ in females during cycling at 70% $\dot{V}$O_2peak_ in 32 °C and 50% RH [50]. Morris et al. [22] also demonstrated that sodium ingestion significantly reduced tympanic temperature by ~0.3 °C, compared with no treatment, in a subanalysis of their cohort (*n* = 7) of trained cyclists during a 60 min constant work rate cycle in 50% RH°C and 18–20% RH (Fig. 6).

During variable intensity exercise, there have been conflicting reports about the effect of hyperhydration on core temperature. Hitchins et al. [58] found no significant differences in core temperature after glycerol hyperhydration compared with control during a 30 min variable power output cycling protocol in which participants were encouraged to ride at maximal intensity in ~33.2 °C and ~57.8% RH. Hillman et al. [60] also found no significant differences in core temperature during a 90 min cycling bout in which participants covered as much distance as possible in 35 °C and 40% RH between glycerol- and water-induced hyperhydration and no treatment. A 48 km mountain bike time-trial field study also found no difference in core temperature between glycerol- and water-induced hyperhydration and no treatment [57]. A similar result was reported after a treadmill running time-trial in 30 °C and 50% RH after glycerol-induced hyperhydration compared with placebo [53]. During a self-paced 60 min time-trial in 34.5 °C and 63.4% RH, Marino et al. [48] reported no significant differences in core temperature between glycerol and placebo. However, Easton et al. [55] reported a 0.25 °C lower core temperature during a 16.1 km time-trial in 30 °C and 70% RH, after constant work rate exercise, with creatine + glycerol supplementation compared with pre-supplementation. A similar finding was reported by Gigou et al. [28] who found that, during a 18 km treadmill running time-trial in 28 °C and 25–30% RH, sodium-induced hyperhydration significantly reduced end-exercise core temperature by 0.3 °C compared with no treatment. No study found a significant difference in skin temperature at any time point between hyperhydration and control during constant work rate exercise [16, 35, 50, 61], variable work rate exercise [28, 48, 60] or protocols that involved a combination of constant and variable work rate [17, 55, 58].

## Discussion

The main finding of this systematic review was that pre-exercise hyperhydration may improve exercise capacity (i.e. time to exhaustion) at a constant work rate. The improvement in exercise capacity appears to be associated with an increase in plasma volume and a subsequent reduction in core temperature and heart rate when exercising at a given work rate. A secondary finding was that hyperhydration may induce gastrointestinal symptoms in some individuals, compared with euhydration, when appropriate mitigation strategies are not implemented. Thirdly, pre-exercise hyperhydration does not appear to improve time-trial performance, although this could be due to methodological considerations within studies (e.g. type of exercise protocol).

### Hyperhydration and Constant Work Rate Exercise to Exhaustion

Hyperhydration appears to improve exercise capacity at a constant work rate, with five of seven studies reporting a significant increase in time to exhaustion (14.3–26.2%) between treatment and control [16, 34, 47, 50, 63]. The improvement in exercise capacity may have been due to an improvement in cardiovascular stability, characterised by an acute increase in plasma volume [16, 50] and a decrease in heart rate [34, 47], along with a reduction in core temperature [16, 50, 63]. Only two of the five studies that found an improvement in exercise capacity measured plasma volume and reported a significant increase of ~4.5–6.3% after a high sodium dose compared with a low sodium dose [16, 50], which does support the premise of improved cardiovascular stability and also presents an area for future investigation. During exercise, sweat is hypotonic relative to plasma, such that more water is lost than solutes [69]. As exercise duration increases, without adequate fluid intake, plasma volume continues to decrease with each percentage of body mass lost [2, 70]. In addition to the concomitant increase in core temperature and heart rate that is seen with hypohydration, the reduction in plasma volume has flow-on effects on extracellular osmolality. Hyperosmolality stimulates the sensation of thirst and may increase perceived exertion and negatively impact overall mood, which may adversely affect exercise performance [71, 72]. If pre-exercise hyperhydration can increase plasma volume, then it may delay these negative effects and attenuate the detrimental effects of hypohydration, providing a performance advantage. An increase in blood volume via hyperhydration could improve venous return during exercise by maintaining mean arterial pressure, which would prevent large decreases in stroke volume during endurance exercise [17]. Maintaining venous return would also contribute to attenuate the increase in heart rate at a given work rate. Indeed, almost all studies that reported an increased time to exhaustion found a significant reduction in mean heart rate after hyperhydration of 3–9 beats min^−1^ [34, 50, 63]. Furthermore, one study reported a decrease in heart rate after hyperhydration of ~6–7 beats min^−1^ during constant work rate exercise (120 min) compared with control [47]. These data demonstrate that hyperhydration can attenuate cardiovascular strain during constant work rate exercise.

Pre-exercise hyperhydration may reduce core temperature during constant work rate exercise. Of the five studies that improved time to exhaustion, two found a significant reduction in end-exercise core temperature [16, 63] and one found a significant decrease in the rate of rise of core temperature [50] after hyperhydration compared with control. The two studies that did not report an attenuated increase in core temperature may be explained by the lower environmental conditions (23–27 °C), although Goulet et al. [47] did report a non-significant trend for core temperature to be lower after hyperhydration. An increase in blood volume, stemming from hyperhydration, may attenuate the rise in core temperature compared to hypohydration, particularly during exercise in hot conditions, where sweat rate is elevated. In hypohydrated participants (~4.9% BM), cardiac output and mean arterial pressure are reduced during constant work rate exercise in hot conditions, as is skin blood flow and sweat rate [7]. A reduction in heat loss via sweating results in an increase in heat storage during exercise in the heat [17]. During passive rest, sodium ingestion has been found to increase plasma volume by ~7.9% compared with glycerol ingestion [29, 30] and ~12.6% compared with water [30]. However, these results conflict with more recent work that found no significant differences between sodium, glycerol and sodium + glycerol ingestion after 180 min of passive rest [15]. The majority of studies have investigated changes in plasma volume during passive rest studies only (Fig. 5), which presents an opportunity to determine the effects of increasing plasma volume prior to constant work rate exercise on the subsequent physiological responses (i.e. heart rate and core temperature). Therefore, future research should investigate the effects of hyperhydration on plasma volume and the subsequent effects on thermoregulation and cardiovascular function in both constant work rate and variable intensity exercise in hot conditions.

### Hyperhydration and Time-Trial Performance

Although the findings of this review suggest that hyperhydration does not improve time-trial performance, methodological differences between studies provide some explanation for this result. For example, Scheadler et al. [53] and Souza et al. [54] utilised relatively short durations of running (~60 min and ~48 min, respectively) and elicited BM losses of 2.4% and 1.6%, respectively, meaning that the time actually spent ≥ 2% BM loss may not have been sufficient to exacerbate physiological strain and impact on performance. Furthermore, the protocol used by Souza et al. [54] involved ad libitum and unmeasured intakes of both the control and sodium hyperhydration treatments, meaning that the treatments were neither standardized nor transparent; this poor methodology may explain the non-significant performance result. Meanwhile, McCullagh et al. [49] implemented a constant work rate period consisting of a 10 km run and 40 km cycle before a 5 km run time-trial and found no difference in performance. However, a limitation of this study was the small sample size (*n* = 6) which was not justified. Gigou et al. [28] implemented the longest running time-trial of 18 km after sodium ingestion; however, participants were stopped at 9 km for 15 min of data collection (urine volume, urine specific gravity and BM) before finishing the final 9 km. This indicates that it was not a true time-trial and may have lacked ecological validity. In this study, sodium ingestion increased pre-exercise fluid retention by 1354 mL (58.5% of fluid retained) compared with no treatment, but there were no significant improvement in performance [28].

In terms of hyperhydration and endurance running, it is important to consider the interaction of changes in BM and hydration status on performance, with hyperhydration having potentially negative effects on running economy (defined as the energy demand for a given submaximal running speed [73]) from the fluid-induced BM gain. Previous research in this area has found that adding external mass to running shoes impairs running economy and exercise performance [74]. Unfortunately, despite the recognition of running economy as one of the predictors of endurance running performance [73], only one study has investigated the increase in BM after hyperhydration on this characteristic. Here, the authors reported no significant differences in running economy at moderate speeds (i.e. 60% $\dot{V}$O_2max_) [65]. Additional investigations are required to determine whether running economy is altered after hyperhydration at typical running speeds for endurance events, such as the marathon (i.e. 80–85% $\dot{V}$O_2max_ [75]).

Although cycling on flat terrain is considered a weight-supported sport and less affected by small changes in BM, some studies of laboratory protocols have provided data on differences in BM that should be considered for their relevance to real-world cycling on hilly courses. In cycling studies involving constant work rate exercise, Easton et al. [55] reported an end-exercise BM loss of 2.3% after glycerol ingestion compared with 2.0% BM loss after placebo. Similarly, Polyviou et al. [56] reported a 1.8% BM loss after creatine + glycerol + glucose ingestion and the same supplementation protocol with the addition of alpha lipoic acid after ~66 min of cycling. Wingo et al. [57] reported no differences in 48 km mountain bike time-trial performance in 28 °C after glycerol ingestion. Wingo et al. [57] compared glycerol with water only and no treatment (indicating that it was not placebo controlled), nor was dietary intake recorded, standardised or analysed in the 24 h prior to the trial. Furthermore, during this field study, participants were stopped after each 16 km loop for 8 min for measures of core temperature, hydration status and blood analysis.

Sodium hyperhydration was found to improve time-trial performance in cyclists after an initial dehydration period. Morris et al. [22] found a significant improvement (11.4%) in a 200 kJ time-trial in 30 °C after 60 min of constant work rate cycling following sodium (60 mg kg^−1^ BM) ingestion. Fluid retention increased after sodium ingestion by ~577 mL compared with placebo and ~673 mL compared with no treatment. In this study, sodium ingestion facilitated the retention of 59.5% of fluid ingested, compared with 26.4% and 18.9% after placebo and no treatment, respectively. After 60 min of constant work rate exercise, BM decreased by 0.7% after sodium ingestion, compared with 1.8% after placebo and 1.9% after no treatment. Therefore, the fluid retained after sodium ingestion may have delayed the negative effects of dehydration during the time-trial with BM losses increasing to 1.4% after sodium ingestion compared with ~2.3% after placebo and no treatment post time-trial. The aforementioned study is limited by its small sample size (*n* = 9) and an apparent lack of control in regard to fluid standardisation between treatments, but indicates that sodium may be a practical osmotic agent to utilise prior to exercise in hot conditions. Future research investigating time-trial performance should continue to investigate a range of exercise modalities, including endurance running and the effect of hyperhydration on running economy. Furthermore, a strong level of control (e.g. dietary standardisation) is required to accurately assess the effect of hyperhydration on exercise performance.

### Hyperhydration and Gastrointestinal Symptoms

Hyperhydration may cause gastrointestinal symptoms compared with euhydration prior to and during exercise when appropriate ingestion strategies are not used. Indeed, ingesting an isolated bolus of glycerol has resulted in ‘minor’ nausea and vomiting [35]; however, it was reported that co-ingesting glycerol with fluid did not elicit any symptoms of discomfort, headaches or gastrointestinal distress [58]. Furthermore, this review found that aliquoting the hyperhydration treatment and bolus into equal doses does not elicit differences in gastrointestinal symptoms, compared with other treatments and a matched volume of fluid ingested [15, 29, 30, 46]. The slower rate of fluid ingestion may be a practical approach for athletes who are training or competing in hot conditions. Athletes utilising this ingestion approach may still complete usual aspects of their pre-training or competition routine (e.g. warm up) whilst completing the hyperhydration protocol and minimising the gastrointestinal symptoms experienced. There may also be a dose–response relationship with gastrointestinal symptoms, increasing with larger amounts of sodium ingested [36]. In terms of comparison between hyperhydrating treatments, no significant differences in gastrointestinal symptoms have been found between sodium chloride and glycerol [15, 29, 30] or sodium bicarbonate and sodium citrate [46]. One study reported no difference in gastrointestinal discomfort between sodium chloride in solution or dissolvable tablet form; however, the latter may improve palatability given the salty taste of sodium [29]. There were also no significant differences in abdominal discomfort between glycerol, sodium and glycerol + sodium ingestion [15]. However, a limitation in this area of hyperhydration research is the method by which gastrointestinal symptoms are quantified. The current review found that early studies relied on participants self-reporting symptoms, foregoing the use of valid or reliable scales or questionnaires [33–35]. Other studies included a post-trial or post-study survey of symptoms [17, 18, 58]. The most common method used was a 1–5 Likert scale [15, 28–30, 47], which does not appear to have been assessed for validity or reliability. Only two studies have used a questionnaire designed to measure the incidence and severity of symptoms in different environmental conditions [46, 57]. Future research in hyperhydration should continue to implement a valid quantitative method of assessing gastrointestinal symptoms to understand the potential negative effects of hyperhydrating. Valid assessment of gastrointestinal symptoms is also required when comparing hyperhydration protocols (e.g. glycerol compared with sodium) to ensure the optimal hyperhydration strategy, which minimises gastrointestinal symptoms and can be implemented by athletes prior to competition or training in hot conditions.

### Limitations and Considerations for Future Research

The findings of this review indicate an underrepresentation of females in hyperhydration research, with only one study solely including female participants [50] and ~90% of participants across included studies in this review being male. There may be differences in key physiological outcomes (i.e. fluid regulation and thermoregulation) between males and females in response to hyperhydration. During the menstrual cycle, basal core temperature can increase by ~0.4 °C [76] and it has been suggested that the risk of females suffering heat-related illness as a result of this elevated core temperature is increased [77]. Indeed, prior to 60 min of cycling (~22 °C and 60% RH), baseline mean core temperature was 0.3 °C higher during the luteal phase compared with the midfollicular phase and this increased to 0.6 °C during exercise [78]. Therefore, appropriate heat-mitigation strategies, such as pre-exercise hyperhydration, require detailed investigation as to how they may impact female thermoregulation. The effectiveness of glycerol hyperhydration during different stages of the menstrual cycle may be adversely affected, given that both estradiol and progesterone have been reported to influence fluid balance and osmoregulation in females [79]. Indeed, some studies included in this review did include female participants in glycerol hyperhydration research, [18, 27, 33, 34, 47–49, 51, 52]; however, no study adequately accounted for the menstrual cycle. The one study that investigated female participants found that exercise capacity increased by 25% in oral contraceptive users and by 26% in naturally menstruating females in the luteal phase after sodium hyperhydration, compared with a low sodium control [50]. A limitation of this protocol is that only 10 mL kg^−1^ BM of fluid was administered during the hyperhydration protocol, which is less than half of the recommended fluid dose for inducing hyperhydration [80]. Future research investigating females should ensure that menstrual cycle phase is appropriately quantified using diaries and/or hormone analysis [81]. Furthermore, a comparison of differing hyperhydrating agents, such as glycerol and sodium across the menstrual cycle, is required to determine the effect of hormone levels on fluid retention during hyperhydration.

Future research in hyperhydration should continue to explore different approaches within the context of endurance exercise in hot conditions. Recent research has demonstrated the ability of a combined ingestion protocol of glycerol + sodium to increase the fluid retained compared to glycerol and sodium ingested alone; however, this was done under resting conditions [15]. It is currently unknown whether the enhanced fluid retention after the ingestion of glycerol + sodium provides significant physiological, or performance benefits compared with glycerol or sodium ingestion alone. Emphasis should also be placed on investigating the effect of hyperhydration on different exercise modalities, with this review finding only a small number of studies that have investigated hyperhydration and endurance running performance, and only one field-based study. The absence of field-based research may be due to the difficulties associated with maintaining a level of control (e.g. environmental conditions) compared with a laboratory setting. The lack of endurance running studies (laboratory and field based) may be due to the possible gastrointestinal symptoms associated with endurance running and hyperhydration inducing greater or more severe gastrointestinal symptoms. However, as previously highlighted, the prevalence and incidence of gastrointestinal symptoms is yet to be adequately quantified with the use of valid and reliable tools. Hyperhydration may also be useful when combined with other heat mitigation strategies, such as pre-cooling. Previous work in this area has demonstrated that combining pre-cooling (ice slurry) with glycerol hyperhydration resulted in a reduction in pre-exercise core temperature by 0.4 °C compared with control [82]. The combined pre-cooling and hyperhydration strategy provided no performance benefit compared with a control (chilled fluid) so this is an area to explore in greater detail.

While it appears that pre-exercise hyperhydration can improve exercise capacity, it is also important to consider the reliability of such tests when compared with time-trials. Typically, time to exhaustion protocols have a high coefficient of variation (CV; > 10%) when compared with time-trials (< 5%) [83], indicating that the reliability of time to exhaustion protocols can be poor. Furthermore, time-trial performance provides a valid test of exercise performance given that it replicates the demands of athletes in real-world competition [83], rather than assessing exercise capacity. With regards to hyperhydration, time to exhaustion protocols are useful for providing a controlled environment (e.g. constant work rate) when assessing physiological measures (i.e. heart rate and core temperature) and the potential mechanisms when comparing treatments (i.e. glycerol or sodium) [83]. However, caution must be applied when assessing the performance effect of time to exhaustion protocols and hyperhydration, and the limited application of such protocols to sporting performance.

Adequate blinding strategies should be implemented when subjective measurements (e.g. gastrointestinal symptoms or perceived exertion) or exercise performance are being assessed. Blinding is important as there are previous reports of a placebo effect in research involving ergogenic aids and exercise performance [84, 85]. Glycerol is a sweet tasting liquid; therefore, the comparator should be matched for taste and volume. Studies involving sodium should use dissolvable tablets and the comparator matched for total number of tablets, filled with an inert substance. Sodium dissolved in a solution may result in greater fluid retention than using dissolvable tablets [29]; however, there are concerns around the palatability of this method [28, 29]. Additionally, control protocols that provide a matched fluid volume may adequately blind participants, although it is recognised that urine volume will differ between treatments. Further investigations should also examine hyperhydrating agents (i.e. glycerol + sodium) in the context of exercise performance to determine the impact of the extra fluid retained, compared with glycerol or sodium alone. Finally, a standardised approach to the assessment of gastrointestinal symptoms is required so that results between studies are comparable.

## Conclusions

This review evaluated the existing literature on the effectiveness of hyperhydration on exercise performance, physiological outcomes and gastrointestinal symptoms. The main finding was that hyperhydration can improve exercise capacity, potentially due to an increase in plasma volume that may aid in reducing heart rate and core temperature when exercising at a constant work rate to exhaustion, although it must be acknowledged that not all studies measured changes in plasma volume which provides scope for further investigation. Future research should investigate the effects of hyperhydration on exercise performance in more practical settings (e.g. field studies or competitive events) using valid and reliable protocols (i.e. time-trials) in thermally challenging conditions using different modalities (e.g. endurance running) with a strong level of control. The effect of different phases of the menstrual cycle on key outcome variables in females (e.g. fluid retention and core temperature) remains to be adequately investigated and represents the next immediate priority area for this research area.

### Supplementary Information

Below is the link to the electronic supplementary material.

Supplementary file1 (DOCX 32 KB)

## Declarations

### Availability of Data and Material

The data sets used to prepare this manuscript are available from the corresponding author on reasonable request.
