# Supplementary material for: The Effect of Pre-Exercise Hyperhydration on Exercise Performance, Physiological Outcomes and Gastrointestinal Symptoms: A Systematic Review
Source: Sports Med. 2023 Jul 25;53(11):2111–34. doi: 10.1007/s40279-023-01885-2 (PMC10587316; doi:10.1007/s40279-023-01885-2)
Supplement: Supplementary file 1 — Supplementary file1 (DOCX 32 KB) [file 40279_2023_1885_MOESM1_ESM.docx]

**Article Name:** The Effect of Pre-Exercise Hyperhydration on Exercise Performance, Physiological Outcomes and Gastrointestinal Symptoms: A Systematic Review

**Journal Name:** Sports Medicine

**Authors:** William T. Jardine^1^, Brad Aisbett^1^, Monica K. Kelly^1^, Louise M. Burke^2^, Megan L. Ross^2^, Dominique Condo^1^, Julien D. Périard^3^, Amelia J. Carr^1^

**Author Affiliations:**

^1^Deakin University, School of Exercise and Nutrition Sciences, Centre for Sport Research, Geelong, Victoria, Australia

^2^Australian Catholic University, Mary MacKillop Institute for Health Research, Exercise and Nutrition Research Program, Watson, Australian Capital Territory, Australia

^3^University of Canberra, Research Institute for Sport and Exercise, Bruce, Australian Capital Territory, Australia

**Corresponding Author:** William T Jardine

Deakin University, School of Exercise and Nutrition Sciences, Centre for Sport Research, Geelong, Victoria

Email: [wjardine@deakin.edu.au](mailto:wjardine@deakin.edu.au)

Phone: +61 (03) 9251 7309

**Electronic Supplementary Material**

| Author and year | | Was the research question clearly stated? | | Was the selection of study subjects/ patients free from bias? | | Were study groups comparable? | | Was method of handling withdrawals described? | | Was blinding used to prevent introduction of bias? | | Were intervention/ therapeutic regimens/exposure factor or procedure and any comparison(s) described in detail? Were the intervening factors described? | | Were outcomes clearly defined and the measurements valid and reliable? | | Was the statistical analysis appropriate for the study design and type of outcome indicators? | | Are conclusions supported by results with biases and limitations taken into consideration? | | Is bias due to study’s funding or sponsorship unlikely? | | Overall rating | |
| --- | --- | --- | --- | --- | --- | --- | --- | --- | --- | --- | --- | --- | --- | --- | --- | --- | --- | --- | --- | --- | --- | --- | --- |
| Anderson et al. 2001 [17] | Y | | U | | N/A | | N/A | | Y | | Y | | Y | | Y | | Y | | Y | | Ø | |  |
| Beis et al. 2011 [65] | Y | | U | | N/A | | Y | | U | | Y | | Y | | Y | | U | | U | | Ø | |  |
| Cian et al. 2000 [66] | Y | | U | | Y | | N/A | | N | | Y | | U | | Y | | U | | U | | Ø | |  |
| Coutts et al. 2002 [18] | Y | | U | | Y | | N/A | | Y | | Y | | Y | | Y | | Y | | Y | | Ø | |  |
| Souza et al. 2018 [54] | Y | | Y | | Y | | N/A | | N | | N | | Y | | Y | | U | | N | | Ø | |  |
| Dini et al. 2007 [59] | Y | | U | | U | | N/A | | U | | Y | | Y | | Y | | Y | | U | | Ø | |  |
| Easton et al. 2007 [55] | Y | | U | | Y | | Y | | Y | | Y | | Y | | Y | | Y | | Y | | Ø | |  |
| Freund et al. 1995 [19] | Y | | U | | Y | | U | | Y | | Y | | Y | | Y | | Y | | U | | Ø | |  |
| Fujii et al. 2021 [68] | Y | | U | | Y | | N/A | | N | | Y | | Y | | Y | | Y | | U | | Ø | |  |
| Gigou et al. 2012 [28] | Y | | U | | N/A | | N/A | | N | | Y | | Y | | Y | | Y | | Y | | Ø | |  |
| Goulet et al. 2002 [63] | Y | | U | | N/A | | N/A | | Y | | Y | | Y | | N/A | | Y | | U | | Ø | |  |
| Goulet et al. 2006 [62] | Y | | U | | Y | | N/A | | Y | | Y | | Y | | Y | | Y | | U | | Ø | |  |
| Goulet et al. 2008 [47] | Y | | U | | Y | | N/A | | Y | | Y | | Y | | Y | | Y | | U | | Ø | |  |
| Goulet et al. 2018 [15] | Y | | U | | Y | | N/A | | N | | Y | | Y | | Y | | Y | | Y | | Ø | |  |
| Hillman et al. 2013 [60] | Y | | U | | Y | | N/A | | Y | | Y | | Y | | Y | | U | | U | | Ø | |  |
| Hitchins et al. 1999 [58] | Y | | U | | Y | | N/A | | Y | | Y | | Y | | Y | | U | | U | | Ø | |  |
| Kilduff et al. 2004 [61] | Y | | U | | Y | | N/A | | Y | | Y | | Y | | Y | | U | | U | | Ø | |  |
| Koehler et al. 2014 [27] | Y | | U | | Y | | N/A | | Y | | Y | | Y | | Y | | Y | | U | | Ø | |  |
| Latzka et al. 1997 [35] | Y | | U | | Y | | N/A | | Y | | Y | | Y | | Y | | U | | U | | Ø | |  |
| Lyons et al. 1990 [33] | Y | | U | | Y | | N/A | | U | | Y | | Y | | U | | U | | U | | Ø | |  |
| Marino et al. 2003 [48] | Y | | U | | Y | | N/A | | Y | | Y | | Y | | Y | | U | | U | | Ø | |  |
| McCullagh et al. 2013 [49] | Y | | U | | Y | | N/A | | Y | | Y | | Y | | Y | | U | | U | | Ø | |  |
| Melin et al. 2002 [67] | Y | | U | | Y | | N/A | | N | | Y | | Y | | Y | | U | | U | | Ø | |  |
| Montner et al. 1996 [34] | Y | | U | | Y | | N/A | | Y | | Y | | Y | | Y | | U | | U | | Ø | |  |
| Montner et al. 1999 [51] | Y | | U | | Y | | N/A | | Y | | Y | | Y | | Y | | U | | U | | Ø | |  |
| Morris et al. 2015 [22] | Y | | U | | Y | | N/A | | Y | | Y | | Y | | Y | | U | | U | | Ø | |  |
| O'Brien et al. 2005 [64] | Y | | U | | Y | | N/A | | Y | | Y | | Y | | Y | | U | | U | | Ø | |  |
| Polyviou et al. 2012 [56] | Y | | U | | Y | | N/A | | Y | | Y | | Y | | Y | | U | | U | | Ø | |  |
| Riedesel et al. 1987 [52] | Y | | U | | Y | | N/A | | U | | Y | | Y | | U | | U | | U | | Ø | |  |
| Savoie et al. 2015 [30] | Y | | U | | Y | | N/A | | Y | | Y | | Y | | Y | | Y | | Y | | Ø | |  |
| Savoie et al. 2016 [29] | Y | | U | | Y | | N/A | | N | | Y | | Y | | Y | | Y | | U | | Ø | |  |
| Scheadler et al. 2010 [53] | Y | | U | | Y | | Y | | Y | | Y | | Y | | U | | Y | | U | | Ø | |  |
| Siegler et al. 2021 [46] | Y | | U | | Y | | N/A | | Y | | Y | | Y | | Y | | Y | | U | | Ø | |  |
| Sims et al. 2007 [16] | Y | | U | | Y | | N/A | | Y | | Y | | Y | | Y | | Y | | U | | Ø | |  |
| Sims et al. 2007 [50] | Y | | U | | Y | | N/A | | Y | | Y | | Y | | Y | | Y | | U | | Ø | |  |
| Sugihara et al. 2014 [36] | Y | | U | | Y | | N/A | | U | | Y | | Y | | Y | | Y | | Y | | Ø | |  |
| Wingo et al. 2004 [57] | Y | | U | | Y | | N/A | | Y | | Y | | Y | | Y | | Y | | U | | Ø | |  |

**Table S1** Quality assessment of studies using the American Dietetic Association Quality Criteria Checklist assessment tool for quantitative research. Y = yes, N = no, N/A = not application, U = unclear. A positive (+) overall rating was given if studies answered Y to questions 2, 3, 6 and 7 and one additional question, a negative (-) overall rating was given if studies answered N to six or more questions, a neutral (Ø) overall rating was given if the answers to questions 2, 3, 6 and 7 do not indicate the study is exceptionally strong.

| Order | Search Terms |
| --- | --- |
| 01 | Water intoxication |
| 02 | Fluid balance OR hyperhydra* OR overhydra* OR hydra* |
| 03 | 01 OR 02 |
| 04 | Athlet* OR physic* OR exercis* OR perform* OR sport* OR compet* |
| 05 | Physiol* OR adapt* OR plasma volume OR sweat rate OR rectal temperature OR core temperature OR skin temperature OR oxygen consumption OR lactate concentration OR subject* OR rating of perceived exertion OR thermal comfort OR thirst OR urine specific gravity OR fluid retention OR plasma osmolality OR urine osmolality OR urine colour |
| 06 | Headach* OR pain OR nausea OR bloating OR diarrhea OR diarrhoea OR stitch |
| 07 | Gastrointestinal OR gastro-intestinal OR GI N3 symptom* OR indicator* OR issue* OR distress OR discomfort OR disturbance OR problem OR side effect* |
| 08 | 06 OR 07 |
| 09 | 03 AND 05 |
| 10 | 03 AND 04 |
| 11 | 03 AND 08 |
| 12 | 09 OR 10 OR 11 |

**Table S2** The electronic search strategy conducted in SPORTSDiscus using the EBSCOHost database.

| Order | Search Terms |
| --- | --- |
| 01 | Fluid balance OR hyperhydra* OR overhydra* OR hydra* |
| 02 | Athlet* OR physic* OR exercise* OR perform* OR sport* OR compet* |
| 03 | Physiol* OR adapt* OR plasma volume OR heart rate OR sweat rate OR rectal temperature OR core temperature OR skin temperature OR oxygen consumption OR lactate concentration OR subject* OR rating of perceived exertion OR thermal comfort OR thirst OR urine specific gravity OR fluid retention OR plasma osmolality OR urine osmolality OR urine colour |
| 04 | Headache* OR pain or nausea OR bloating OR diarrhea OR diarrhoea OR stitch |
| 05 | Gastrointestinal OR gastro-intestinal OR GI N3 symptom* OR indicator* OR issue* OR distress OR discomfort OR disturbance* OR problem* or side effect* |
| 06 | 04 OR 05 |
| 07 | 01 AND 02 |
| 08 | 01 AND 03 |
| 09 | 01 AND 06 |
| 10 | 07 OR 08 OR 09 |

**Table S3** The electronic search strategy conducted in Medline Complete.

| Order | Search Terms |
| --- | --- |
| 01 | Hyperhydration/exp |
| 02 | Fluid balance OR hyperhydr* OR overhydra* OR hydra* |
| 03 | 01 OR 02 |
| 04 | Athlete* OR physic* OR exercise* OR perform* OR sport* OR compet* |
| 05 | Physiol* OR adapt* OR plasma volume OR heart rate OR sweat rate OR rectal temperature OR core temperature OR skin temperature OR oxygen consumption OR lactate concentration OR subject* OR rating of perceived exertion OR thermal comfort OR thirst OR urine specific gravity OR fluid retention OR plasma osmolality OR urine osmolality OR urine colour |
| 06 | Headache* OR pain OR nausea OR bloating OR diarrhea OR diarrhoea OR stitch |
| 07 | Gastrointestinal OR gastro-intestinal OR GI NEAR/3 symptom* OR indicator* OR issue* OR distress OR discomfort OR disturbance* OR problem* OR side effect* |
| 08 | 06 OR 07 |
| 09 | 03 AND 04 |
| 10 | 03 AND 05 |
| 11 | 03 AND 08 |
| 12 | 09 OR 10 OR 11 |

**Table S4** The electronic search strategy conducted in Embase.
